# Platycodon saponins from Platycodi Radix (*Platycodon grandiflorum*) for the Green Synthesis of Gold and Silver Nanoparticles

**DOI:** 10.1186/s11671-018-2436-2

**Published:** 2018-01-17

**Authors:** Yoonho Choi, Sehyeon Kang, Song-Hyun Cha, Hyun-Seok Kim, Kwangho Song, You Jeong Lee, Kyeongsoon Kim, Yeong Shik Kim, Seonho Cho, Youmie Park

**Affiliations:** 1 College of Pharmacy, Inje University, 197 Inje-ro, Gimhae, Gyeongnam, 50834 Republic of Korea; 2 Department of Naval Architecture and Ocean Engineering, Seoul National University, 1 Gwanak-ro, Gwanak-gu, Seoul, 08826 Republic of Korea; 3 Offshore Plant Research Division, Korea Research Institute of Ships and Ocean Engineering 32, 1312 Beon-gil, Yuseong-daero, Yuseong-gu, Daejeon, 34103 Republic of Korea; 4 College of Pharmacy and Natural Products Research Institute, Seoul National University, 1 Gwanak-ro, Gwanak-gu, Seoul, 08826 Republic of Korea; 5 Department of Pharmaceutical Engineering, Inje University, 197 Inje-ro, Gimhae, Gyeongnam, 50834 Republic of Korea

**Keywords:** Platycodi Radix, *Platycodon grandiflorum*, Platycodon saponins, Platycodin D, Green synthesis, Gold nanoparticles, Silver nanoparticles, Catalytic activity, Curvature-dependent evolution, AFM images

## Abstract

A green synthesis of gold and silver nanoparticles is described in the present report using platycodon saponins from Platycodi Radix (*Platycodon grandiflorum*) as reducing agents. Platycodin D (PD), a major triterpenoidal platycodon saponin, was enriched by an enzymatic transformation of an aqueous extract of Platycodi Radix. This PD-enriched fraction was utilized for processing reduction reactions of gold and silver salts to synthesize gold nanoparticles (PD-AuNPs) and silver nanoparticles (PD-AgNPs), respectively. No other chemicals were introduced during the reduction reactions, providing an entirely green, eco-friendly, and sustainable method. UV-visible spectra showed the surface plasmon resonance bands of PD-AuNPs at 536 nm and PD-AgNPs at 427 nm. Spherically shaped nanoparticles were observed from high-resolution transmission electron microscopy with average diameters of 14.94 ± 2.14 nm for PD-AuNPs and 18.40 ± 3.20 nm for PD-AgNPs. Minor triangular and other polygonal shapes were also observed for PD-AuNPs along with spherical ones. Atomic force microscopy (AFM) images also demonstrated that both nanoparticles were mostly spherical in shape. Curvature-dependent evolution was employed to enhance the AFM images and precisely measure the sizes of the nanoparticles. The sizes were measured as 19.14 nm for PD-AuNPs and 29.93 nm for PD-AgNPs from the enhanced AFM images. Face-centered cubic structures for both nanoparticles were confirmed by strong diffraction patterns from high-resolution X-ray diffraction analyses. Fourier transform infrared spectra revealed the contribution of –OH, aromatic C=C, C–O, and C–H functional groups to the synthesis. Furthermore, the catalytic activity of PD-AuNPs was assessed with a reduction reaction of 4-nitrophenol to 4-aminophenol in the presence of sodium borohydride. The catalytic activity results suggest the potential application of these gold nanoparticles as catalysts in the future. The green strategy reported in this study using saponins as reducing agents will pave new roads to develop novel nanomaterials with versatile applications.

## Background

With increasing sustainability issues, green chemistry has been a focus in many areas of research. The use of natural products in the synthesis of metallic nanoparticles (MNPs) has attracted considerable interest owing to the sustainability of these methods. MNPs have shown versatile applications in material chemistry, biology, and medicine [1–4]. MNPs are generally synthesized by chemical methods through metal ion reduction reactions. Chemical reduction reactions commonly require noxious and toxic chemicals, such as sodium borohydride, to synthesize MNPs. Currently, natural products can replace the use of noxious chemicals and display the following advantages: (i) the synthetic process diminishes noxious chemical wastes; (ii) the green synthetic strategy protects our health and global environment; (iii) the strategy fulfills the overall sustainable initiatives; (iv) the synergistic activities by combining both materials (natural products and MNPs) can be anticipated with increased biocompatibility, which is very beneficial to in vitro and in vivo systems; (v) the strategy is cost effective and amenable to scale-up; and finally, (vi) the green synthetic process can be performed by a one-pot reaction.

AuNPs have been widely applied in the fields of catalysis, drug delivery, chemical and biological sensing, imaging, photothermal therapy, and photodynamic therapy [1, 5–8]. Among various applications, the application as a catalyst in chemical reactions is a gradually growing field. To explore new catalytic applications of AuNPs, a model reaction reducing 4-nitrophenol (4-NP) to 4-aminophenol (4-AP) in the presence of excess sodium borohydride is commonly employed. One of the reasons to use the 4-NP to 4-AP reduction reaction as a model reaction is that the reaction progress can be followed directly by UV-visible spectrophotometry. Without the purification and identification of the final product (i.e., 4-AP), the observation of absorbance changes in the reaction mixture sufficiently demonstrates the reaction progress. AgNPs have been reported to have potent antimicrobial activity. In particular, the high surface-area-to-volume ratio of AgNPs is beneficial to exert antimicrobial activity when compared to their bulk counterparts. AgNPs in the forms of ointments, creams, and gels have been applied in the treatment of burn wounds [9].

Diverse natural products, including amino acids, peptides, fungi, bacteria, plant extracts, algae, polysaccharides, and yeast, have been adapted to serve in green synthesis [10, 11]. Plant extracts have been effectively applied as green-reducing agents for the synthesis of AuNPs and AgNPs [11, 12]. Among diverse plant extracts, traditional Chinese medicines (TCMs) with biological and pharmacological activities are very attractive in the green synthesis of MNPs.

**Fig. 1:**
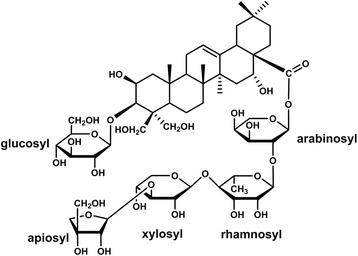
Structure of platycodin D

In the present report, platycodon saponins from Platycodi Radix (*Platycodon grandiflorum*, Campanulaceae) were utilized for the synthesis of AuNPs and AgNPs. Pharmacological activities of Platycodi Radix as a TCM include apothegmatic and antitussive, immunostimulatory, anti-inflammatory, anti-oxidant, anti-tumor, antidiabetic, anti-obesity, hepatoprotective, analgesic, cognitive-enhancing, anticholinergic, and antihistaminic activities [13]. The chemical constituents of Platycodi Radix have been known to contain carbohydrates, proteins, lipids, and triterpenoidal saponins [14]. Triterpenoid saponins belong to a large group of compounds arranged in a four- or five-ring configuration of 30 carbons with several hydroxyl and glycosyl groups, resulting in one end of the molecule being hydrophilic and the other end being hydrophobic [15]. The aglycone of platycodon saponins are oleanane-type triterpenes with two side-chains. A glucose unit is linked to the C-3 position of the triterpene through ether linkage, and diverse glycosyl groups are linked through an ester linkage at the C-28 position. The conjugated glycosyl groups are composed of D-glucose, D-rhamnose, D-arabinose, D-xylose, and D-apiose [16]. Among the platycodon saponins, platycodin D (PD, Fig. 1) is one of the marker compounds of Platycodi Radix. Although PD is one of the major components of triterpenoidal saponins, the total saponin content in Platycodi Radix is approximately 2%. Thus, we developed the enzymatic transformation of platycoside E and platycodin D3 to platycodin D and successfully obtained a PD-enriched fraction from the aqueous extract of Platycodi Radix [16, 17].

In the present report, the PD-enriched fraction was used as a green reducing agent for the synthesis of AuNPs and AgNPs (referred to hereafter as PD-AuNPs and PD-AgNPs). The reaction progress of the synthesis and the surface plasmon resonance (SPR) of each nanoparticle sample was followed by UV-visible spectrophotometry. The sizes and morphologies were observed by microscopic methods, including high-resolution transmission electron microscopy (HR-TEM) and atomic force microscopy (AFM). High-resolution X-ray diffraction (HR-XRD) patterns were obtained to reveal the crystalline structures. Fourier transform infrared (FT-IR) spectra were acquired to elucidate the functional groups that were involved in the synthesis of both nanoparticles. The catalytic activity of the PD-AuNPs was assessed with the reduction reaction of 4-NP to 4-AP in the presence of excess sodium borohydride. Moreover, to precisely identify the nanoscale geometry from the AFM height images, we developed a curvature-dependent evolution scheme that can enhance the surface geometry [18]. The surface evolution equation using the principal curvature flows smooths and enhances the AFM images in the corresponding principal directions. The principal curvatures are computed directly from the first and second derivatives of the discrete AFM height data. Lu et al. studied the effects of curvature flows on morphological features and showed that, while the mean curvature flow could create unwanted new morphological features, no feature points are created under principal curvature flows [19].

## Methods/Experimental

### Materials and Instruments

The PD-enriched fraction from the aqueous extract of Platycodi Radix was prepared by an enzymatic transformation according to our previous report [16, 17]. Hydrochloroauric acid trihydrate (HAuCl_4_·3H_2_O), silver nitrate, sodium borohydride, and 4-nitrophenol were obtained from Sigma-Aldrich (St. Louis, MO, USA). SPR of the nanoparticles and the progress of the 4-NP reduction reaction were followed by a Shimadzu UV-2600 (Shimadzu Corporation, Kyoto, Japan). A JEM-3010 instrument operating at 300 kV was utilized to acquire the HR-TEM images to investigate the sizes and morphologies of the products (JEOL, Tokyo, Japan). To obtain the HR-TEM images, carbon-coated copper grids (carbon type-B, 300 mesh) were purchased from Ted Pella (Redding, CA, USA). A Dimension® Icon® instrument operating with a tapping mode was conducted to obtain the AFM images (Bruker Nano, Santa Barbara, CA, USA). For sample loading, mica (grade V-1, 25 mm × 25 mm length, 0.15 mm thick) was obtained from SPI Supplies Division of Structure Probe (West Chester, PA, USA). A premium high-resolution tapping mode silicon probe (RTESP AFM probe, MPP-11100-10) was purchased from Bruker Nano (Santa Barbara, CA, USA). To elucidate the crystalline structures, a Bruker D8 Discover high-resolution X-ray diffractometer was utilized, which was equipped with a Cu Kα radiation source (λ = 0.154056 nm) (Bruker, Karlsruhe, Germany). The HR-XRD pattern was acquired in the range from 20° to 90° (2θ scale). A KBr pellet was prepared to obtain the FT-IR spectra with a Nicolet 6700 spectrometer in the wavenumber range of 400 ~ 4000 cm^−1^ (Thermo Fisher Scientific, Waltham, MA, USA). For the HR-XRD and FT-IR analyses, an FD5505 freeze-drier was operated for preparing the powdered samples (Il Shin Bio, Seoul, Korea).

### Green Synthesis of PD-AuNPs and PD-AgNPs

A 1 mL sample of PD-AuNPs was synthesized with a final concentration of the PD-enriched fraction (0.05%) and HAuCl_4_·3H_2_O (0.2 mM). The reaction mixture was incubated at ambient temperature for 5 min. A 1 mL sample of PD-AgNPs were synthesized with a final concentration of the PD-enriched fraction (0.01%) and AgNO_3_ (0.8 mM). The reaction mixture was incubated in an 80 °C oven for 3 h and was further incubated at ambient temperature for 21 h. UV-visible spectra were acquired over a range from 300 to 700 nm.

### Curvature-Dependent Evolution for Enhanced AFM Images to Precisely Measure Size

The following curvature flow equation was utilized together with the experimental AFM height data to precisely measure the size and to effectively count the number of nanoparticles.

$$ {\Phi}_{,t}\left(x,y,t\right)=\beta \sqrt{1+{\Phi}_{,x}^2+{\Phi}_{,y}^2=\beta \left|\nabla \Phi \right|} $$, where surface ***S*** = {(*x*, *y*, *z*) : *z* = Φ(*x*, *y*, *z*)}.

If *β* is chosen to be dependent upon the principal curvatures, this evolution process is called a “*curvature flow*.” When *β* is selected as one of the principal curvatures, the corresponding flow is called the *κ*_*i*_ flow (*i* = 1,2). The principal curvature flow makes the images smooth in the corresponding principal direction.

### Catalytic Activity of PD-AuNPs

For the catalytic activity, PD-AuNPs were synthesized as follows: the PD-enriched fraction (0.1%, 500 μL) was mixed with deionized water (480 μL) followed by the addition of HAuCl_4_·3H_2_O (10 mM, 20 μL). The reaction mixture was vortexed for 10 s and incubated at ambient temperature for 24 h in the dark. The catalytic activity of PD-AuNPs was assessed using the 4-NP to 4-AP reduction reaction in the presence of excess sodium borohydride in an aqueous system. The 4-NP solution (900 μL, 0.5 mM) was mixed with deionized water (650 μL). To this solution, freshly prepared sodium borohydride (1.65 mL, 10 mM) was added. Next, freshly synthesized PD-AuNPs (800 μL) were added. The final concentrations of the reaction mixture were as follows for the catalytic activity: 4-NP (0.113 mM, 1 equiv.), sodium borohydride (4.13 mM, 36.5 equiv.), and PD-AuNPs (0.04 mM, 0.354 equiv.). The reaction progress was monitored for 720 s with UV-visible spectrophotometry in the range from 200 to 700 nm at ambient temperature.

## Results and Discussion

### Green Synthesis of PD-AuNPs and PD-AgNPs

**Fig. 2:**
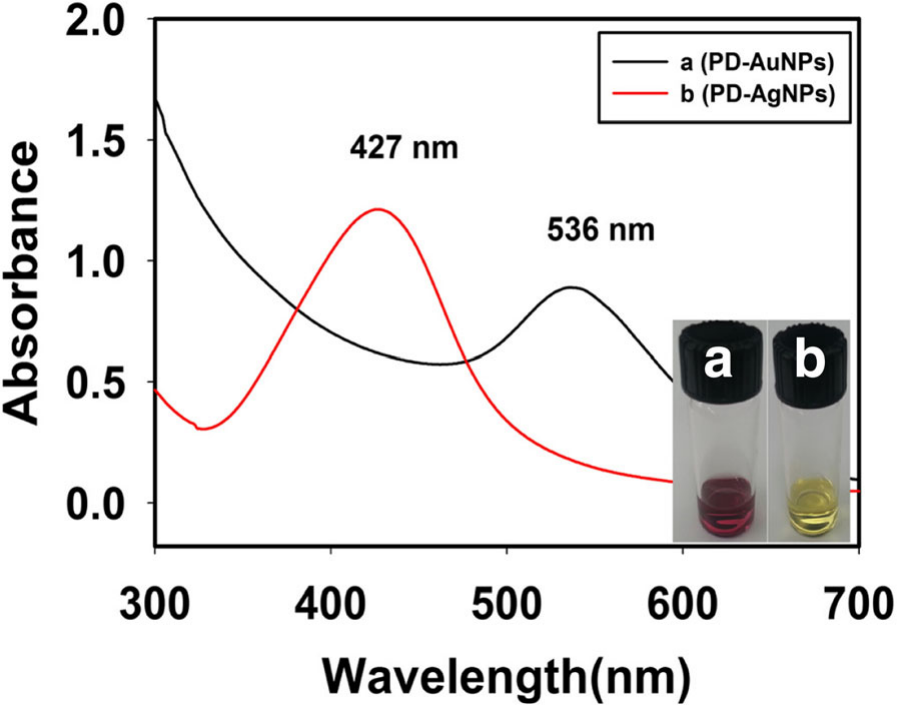
UV-Visible spectra. **a** PD-AuNPs and **b** PD-AgNPs

First, for the synthesis of AuNPs and AgNPs, the reaction completion was easily determined by the visible color changes of the solutions. The color of the PD-AuNPs was wine-purple with a SPR at 536 nm (Fig. 2a). The SPR of PD-AgNPs, which displayed a yellow color, was observed at 427 nm (Fig. 2b). The digital photographs in Fig. 2 show the solutions of PD-AuNPs (left, a) and PD-AgNPs (right, b), which were synthesized according to the procedure described in the previous section. These color changes match the oscillation frequency of conduction electrons in the nanoparticles to the frequency of the incident radiation. Thus, the UV-visible spectra provide sufficient information to determine the reaction completion of AuNPs and AgNPs with their characteristic SPR bands. From the UV-visible spectra shown in Fig. 2, the PD-enriched fraction played a role as a reducing agent to produce both nanoparticles.

### HR-TEM Images

**Fig. 3:**
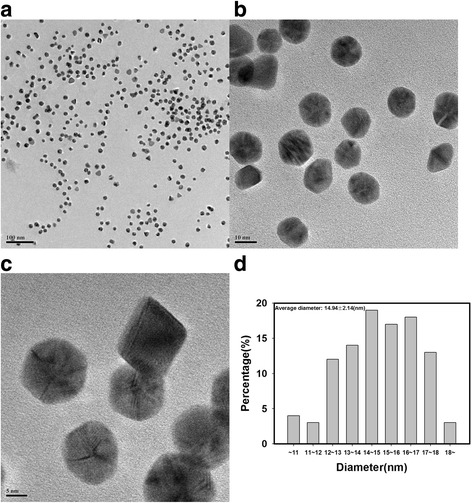
**a**–**c** HR-TEM images and **d** a size histogram of PD-AuNPs. The scale bars represent **a** 100 nm, **b** 10 nm, and **c** 5 nm

**Fig. 4:**
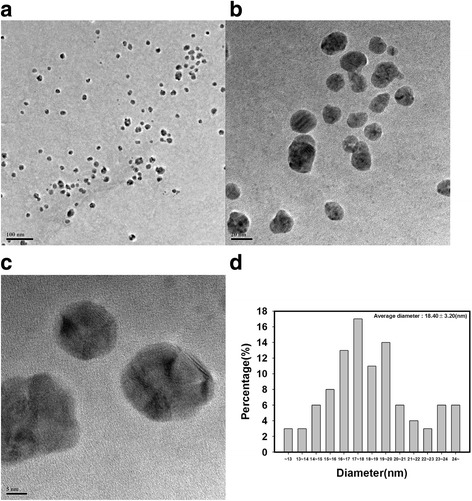
**a–c** HR-TEM images and **d** a size histogram of PD-AgNPs. The scale bars represent **a** 100 nm, **b** 20 nm, and **c** 5 nm

Visualization of the nanoparticles is a crucial step to identifying important information, including size, morphology, and dispersion state. As shown in Fig. 3, PD-AuNPs were spherically shaped with an average diameter of 14.94 ± 2.14 nm. Minor triangular and other polygonal shapes were also observed for PD-AuNPs along with spherical ones. The average diameter of the spherical shapes was measured from 103 discrete nanoparticles from the HR-TEM images. A Gaussian histogram for the distribution of size was observed as Fig. 3d. The most frequently observed size of PD-AuNPs was in the range of 14 ~ 15 nm. As shown in Fig. 3a, PD-AuNPs were well dispersed without any aggregation suggesting that the PD-enriched fraction also acted as a capping agent (or a stabilizing agent). The spherically shaped PD-AgNPs were also observed in Fig. 4. Similar to the dispersion state of PD-AuNPs, the dispersion state of PD-AgNPs was excellent and demonstrated an average diameter of 18.40 ± 3.20 nm (Fig. 4d). One hundred discrete nanoparticles from the HR-TEM images were randomly selected to obtain the average diameter. The most frequently observed size of the PD-AgNPs was in the range of 17 ~ 18 nm.

### AFM Images

**Fig. 5:**
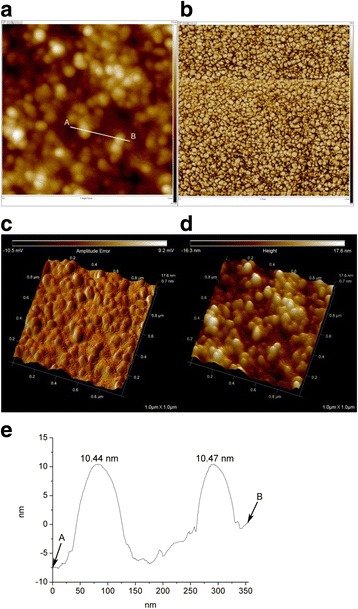
AFM images and section analysis of PD-AuNPs. **a** 2D height (1 μm × 1 μm). **b** 2D phase (2.5 μm × 2.5 μm). **c** 3D amplitude error (1 μm × 1 μm). **d** 3D height (1 μm × 1 μm). **e** Section analysis of line A–B in **a**

The AFM images corroborated well with the HR-TEM images described in the previous section. The spherical morphology of the PD-AuNPs was observed in Fig. 5. In both the 2D height (Fig. 5a) and 3D height (Fig. 5d) images, the brighter nanoparticles possessed higher heights. In addition to topographic information, phase images commonly detect the surface structure, differentiate regions of softness/hardness, and map the different components in materials. As demonstrated in the 2D phase image (Fig. 5b), the spherically shaped PD-AuNPs were well visualized. Additionally, the 3D amplitude error image (Fig. 5c) revealed a spherical morphology. The section analysis was performed, and the result is displayed in Fig. 5e. The line A–B in Fig. 5a was analyzed, and the heights of the two PD-AuNPs were measured as 10.44 and 10.47 nm.

**Fig. 6:**
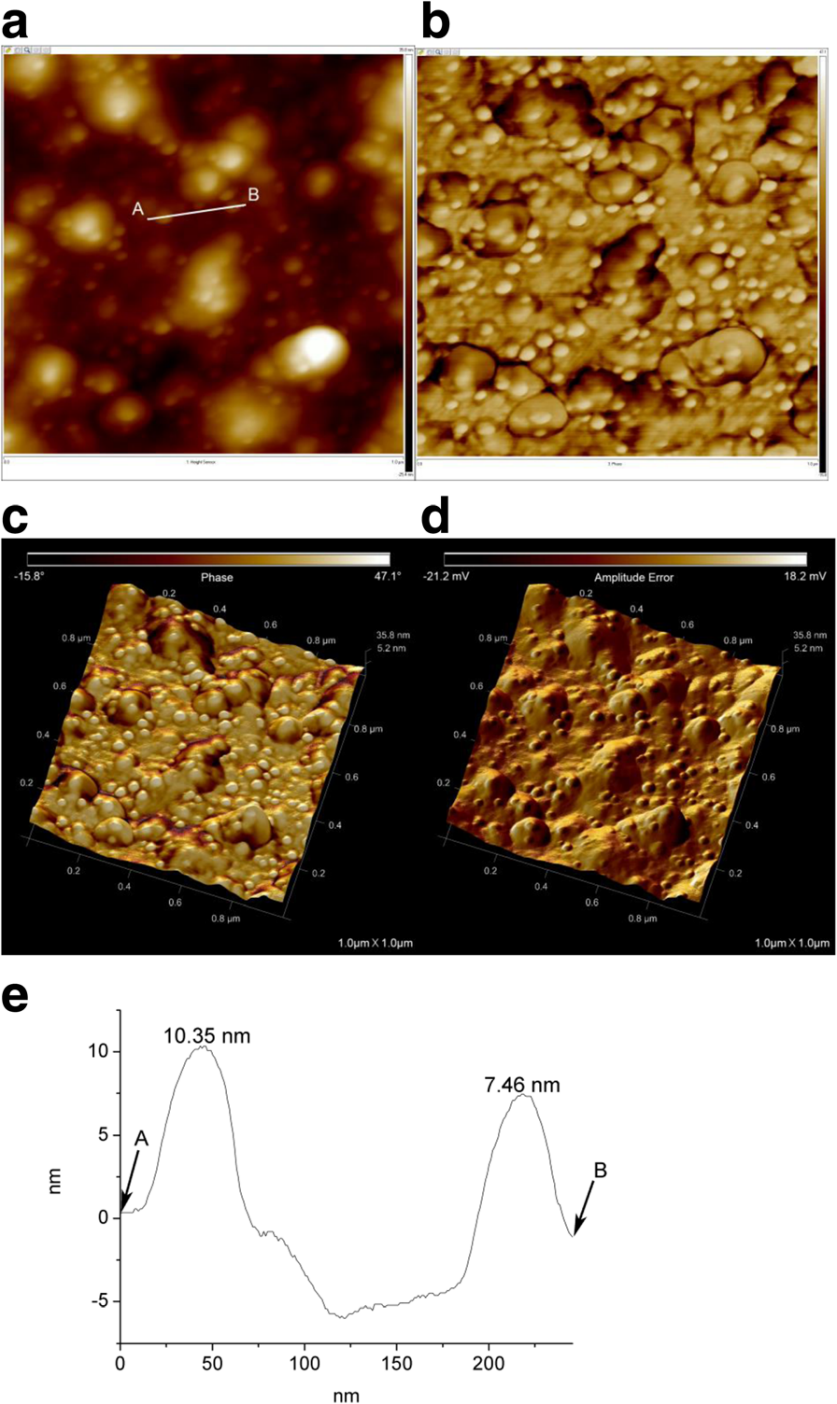
AFM images and section analysis of PD-AgNPs. **a** 2D height (1 μm × 1 μm). **b** 2D phase (1 μm × 1 μm). **c** 3D phase (1 μm × 1 μm). **d** 3D amplitude error (1 μm × 1 μm). **e** Section analysis of line A-B in **a**

Spherically shaped PD-AgNPs were clearly visualized in the 2D height image (Fig. 6a). From the 2D phase (Fig. 6b) and 3D phase (Fig. 6c) images, we observed detailed information regarding the two different components (PD-AgNPs and reducing agents). The brightly colored and spherically shaped materials (that is, PD-AgNPs) retained relatively more hardness than the darker-colored components. The darker-colored components were from the reducing agents (that is, PD-enriched fraction). The spherical morphology of the PD-AgNPs was also confirmed from the 3D amplitude error image (Fig. 6d). The section analysis was also performed and shown in Fig. 6e. The line A–B in Fig. 6a was analyzed, and the height of the two PD-AgNPs was measured as 7.46 and 10.35 nm.

### Curvature-Dependent Evolution for Enhanced AFM Images to Precisely Measure Size

**Fig. 7:**
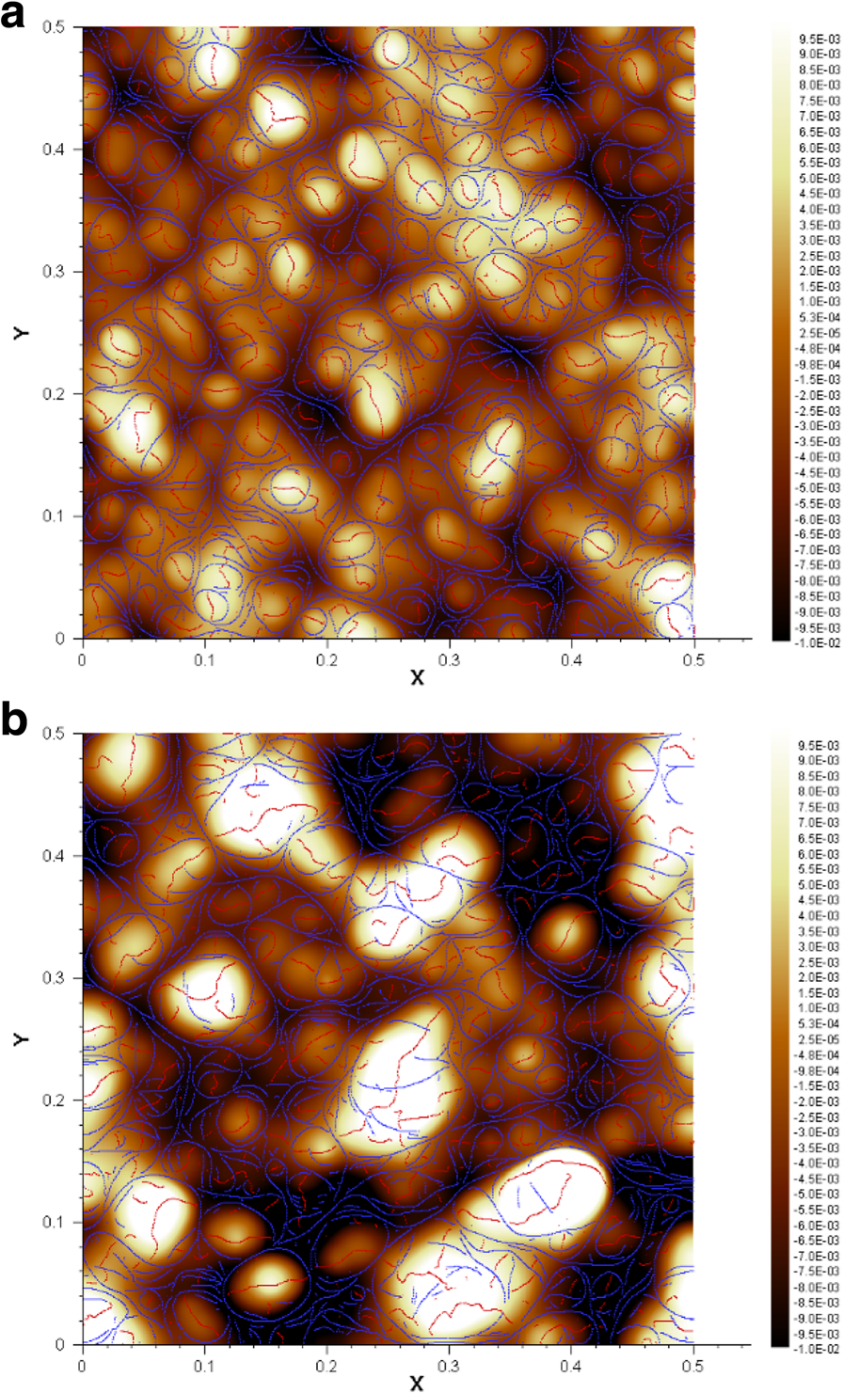
Enhanced AFM 2-D height images by curvature-dependent evolution. **a** PD-AuNPs. **b** PD-AgNPs

Figures 5 and 6 display the 2D and 3D AFM raw data of PD-AuNPs and PD-AgNPs, respectively. From the current height images in Figs. 5a and 6a, precisely identifying the boundary of the nanoparticles is difficult without the phase information in Fig. 5b and Fig. 6b. The 3D images help identify the morphology of the nanoparticles but do not provide the precise sizes of the nanoparticles. Thus, the curvature-dependent evolution with *κ*_2_ flow was employed to identify the valley lines between the nanoparticles and the substrate. As shown in Fig. 7, using a step size of Δ*t* = 10^−7^, 500 evolution steps were performed for the 2D height data of the PD-AuNPs (Fig. 5a) and PD-AgNPs (Fig. 6a). The *κ*_2_ flow precisely identified the major valley lines representing the boundaries of the PD-AuNPs (Fig. 7a) and PD-AgNPs (Fig. 7b). Blue and red lines represented the obtained valley and ridge lines, respectively. From these enhanced images, 30 discrete nanoparticles were selected from each image for the size measurement. The sizes were measured as 19.14 nm for PD-AuNPs and 29.93 nm for PD-AgNPs from the enhanced AFM 2D images. The sizes from the AFM images were larger than those measured in the HR-TEM images (14.94 nm for PD-AuNPs; 18.40 nm for PD-AgNPs). The cold-welding phenomena of the AuNPs on the AFM mica substrate can explain the size discrepancy between the HR-TEM and AFM size measurements [20].

### HR-XRD Analyses

**Fig. 8:**
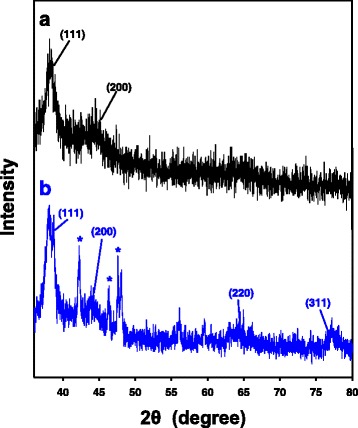
HR-XRD analyses. **a** PD-AuNPs. **b** PD-AgNPs

HR-XRD analysis is required to identify the crystalline structures of the nanoparticles. As demonstrated in Fig. 8, the HR-XRD analyses showed Bragg reflections of the PD-AuNPs and PD-AgNPs, indicating that both types of nanoparticles possessed a face-centered cubic structure. The (111) and (200) planes appeared at 38.2° and 44.4°, respectively in the PD-AuNPs (Fig. 8a). For the PD-AgNPs, the strong diffraction peaks at 38.2°, 44.4°, 65.2°, and 78.0° corresponded to the (111), (200), (220), and (311) planes of the crystalline structure (Fig. 8b). The impurities are marked with asterisks. The (111) plane was the most intense in the HR-XRD patterns of both nanoparticles, indicating that the major orientations of the crystals were along the (111) plane. Next, rough size estimations of both nanoparticles were performed by using the Scherrer equation. Because the (111) peak was the most intense, we estimated the size based on this peak. The definition of each term in the Scherrer eq. (*D* = 0.89 × λ/*W* × cosθ) is as follows: *D* is the particle size, θ is the Bragg diffraction angle of the (111) peak, λ is the X-ray wavelength, and β is the full width at half maximum (FWHM) of the (111) peak in radians. The rough size estimations from the equation resulted in 11.05 nm for the PD-AuNPs and 12.54 nm for PD-AgNPs.

### FT-IR Spectra

**Fig. 9:**
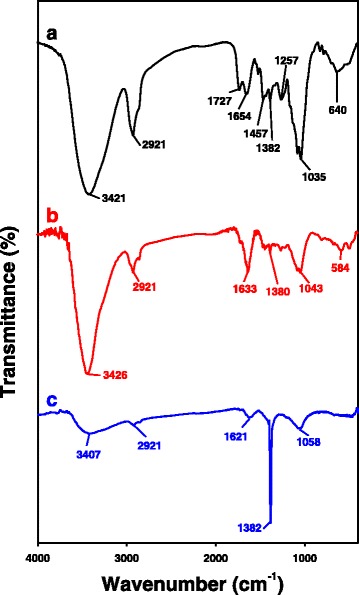
FT-IR spectra. **a** PD-enriched fraction. **b** PD-AuNPs. **c** PD-AgNPs

FT-IR spectra provide important information about which functional groups of the reducing agents were involved in the synthesis of AuNPs and AgNPs. PD is composed of a triterpene aglycone and sugars to form glycosides (Fig. 1). Three FT-IR spectra are shown in Fig. 9: the PD-enriched fraction (Fig. 9a), PD-AuNPs (Fig. 9b), and PD-AgNPs (Fig. 9c). A broad band corresponding to the –OH groups of the PD-enriched fraction appeared at 3421 cm^−1^ (Fig. 9a). Due to hydrogen bonding of the –OH groups, a broad band was observed. This band shifted to 3426 cm^−1^ for PD-AuNPs (Fig. 9b) and 3407 cm^−1^ for PD-AgNPs (Fig. 9c), suggesting that hydroxyl groups were involved in the synthesis. The bands at 1654 cm^−1^ and 1457 cm^−1^ appeared due to aromatic C=C bond vibrations in the PD-enriched fraction (Fig. 9a). After the synthesis, the band at 1654 cm^−1^ shifted to lower wavenumbers, e.g., 1633 cm^−1^ for PD-AuNPs (Fig. 9b) and 1621 cm^−1^ for PD-AgNPs (Fig. 9c). The C–O and C–H vibrations appeared at 1035 cm^−1^ (Fig. 9a), and this band shifted to higher wavenumbers, e.g., 1043 cm^−1^ for PD-AuNPs (Fig. 9b) and 1058 cm^−1^ for PD-AgNPs (Fig. 9c). From the FT-IR results, the –OH, aromatic C=C, C–O, and C–H functional groups in the PD-enriched fraction contributed to the synthesis.

### Catalytic Activity of PD-AuNPs

**Fig. 10:**
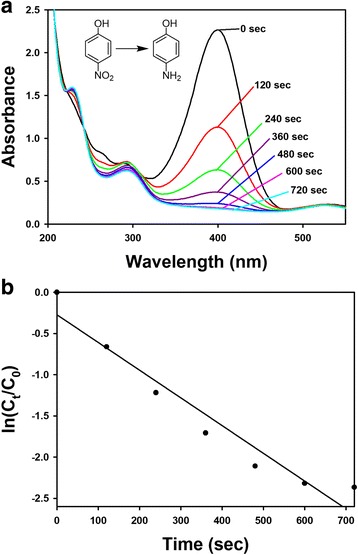
4-NP reduction reaction by sodium borohydride in the presence of the PD-AuNP catalyst. **a** UV-visible spectra and **b** plot of ln(*C*_*t*_/*C*_*0*_) as a function of time (min)

Green-synthesized AuNPs have been successfully applied as a catalyst for 4-NP reduction reaction [21–25]. The catalytic activity of PD-AuNPs was assessed using the reduction reaction of 4-NP to 4-AP in the presence of sodium borohydride. One of major reasons for using the reduction reaction of 4-NP to assess the catalytic activity of AuNPs is that it is very amenable to monitor the reaction by UV-visible spectrophotometry, both qualitatively and quantitatively. The final concentrations of the reaction mixture were as follows for the catalytic activity: 4-NP (0.113 mM, 1 equiv.), sodium borohydride (4.13 mM, 36.5 equiv.), and PD-AuNPs (0.04 mM, 0.354 equiv.). In the presence of excess sodium borohydride (36.5 equiv. to the substrate 4-NP), 4-NP exhibited a maximum absorbance at 400 nm due to the formation of the 4-nitrophenolate anion (data not shown). The color of the 4-nitrophenolate anion solution is yellow, and the reduction reaction did not proceed without the addition of the catalyst. The absorbance at 400 nm did not change until PD-AuNPs were added as a catalyst. As soon as PD-AuNPs were added, the absorbance at 400 nm started to diminish. Interestingly, a new peak at 300 nm appeared simultaneously, which indicated the final product, 4-AP (Fig. 10a). The reaction completed within 720 s in the presence of excess sodium borohydride. We used excess sodium borohydride during the reaction to ensure pseudo-first order kinetics. From the plot of time (sec) and ln(*C*_*t*_*/C*_*0*_) (*C*_*t*_: concentration of 4-NP at 400 nm at time *t*, *C*_*0*_: concentration of 4-NP at 400 nm at time *0*), a linear relationship was observed with a rate constant of 3.4 × 10^−3^/s (Fig. 10b). We could substitute *C*_*t*_ and *C*_*0*_ with *A*_*t*_ and *A*_*0*_, respectively, where *A*_*t*_ is the absorbance at 400 nm at time *t*, and *A*_*0*_ is the absorbance at 400 nm at time *0*. Based on the results, the PD-AuNPs effectively catalyzed the 4-NP reduction reaction to produce 4-AP in the presence of excess sodium borohydride.

In our laboratory, various concentrations of caffeic acid were used for the synthesis of AuNPs, and their catalytic activity was evaluated using 4-nitrophenol reduction reaction [26]. Caffeic acid is one of the secondary metabolites and phenolic compounds found in plants. The results demonstrated that the lowest concentration of caffeic acid exhibited the highest catalytic activity. Additionally, the removal of caffeic acid from the original colloidal solution by centrifugation enhanced the catalytic activity up to 6.41-fold. In the current system, the rate constant of PD-AuNPs was observed to be 3.4 × 10^−3^/s. Possibly the removal of PD after the synthesis of PD-AuNPs by centrifugation can increase the catalytic activity. This will be one of our future work. Both caffeic acid and PD are secondary metabolites from plants and the resulting AuNPs exhibited excellent catalytic activities. Therefore, diverse secondary metabolites of plants can be efficient candidates for green reducing agents to produce AuNP nanocatalysts.

## Conclusions

PD is a major platycodon saponin in Platycodi Radix and is known to possess beneficial biological activities. In the current report, the PD-enriched fraction was employed as a green reducing agent for the synthesis of PD-AuNPs and PD-AgNPs. HR-TEM and AFM images provided information about the size and morphology. Both nanoparticles were mostly spherical with face-centered cubic structures. Curvature-dependent evolution was employed to smooth and enhance the AFM images, allowing the precise measurement of the size. The –OH, aromatic C=C, C–O, and C–H functional groups served as reducing agents to produce the nanoparticles. Moreover, PD-AuNPs displayed catalytic activity toward the 4-NP reduction reaction, suggesting that PD-AuNPs can be applied as a catalyst in the future. Plant metabolites have their own valuable biological activities that, together with the intrinsic activities of the NMPs, frequently show synergistic properties. Thus, one of our future work includes the evaluation of the biological activities of both nanoparticles with in vitro and in vivo studies. In conclusion, the use and expansion of plant metabolites such as saponins in producing novel nanomaterials will continue to increase.
